# Atheroprotective Effects and Molecular Mechanism of Berberine

**DOI:** 10.3389/fmolb.2021.762673

**Published:** 2021-11-16

**Authors:** Lu Xing, Xin Zhou, Ai-Hong Li, Hui-Jin Li, Chun-Xia He, Wei Qin, Dong Zhao, Peng-Quan Li, Li Zhu, Hui-Ling Cao

**Affiliations:** ^1^ Shaanxi Key Laboratory of Ischemic Cardiovascular Disease, Institute of Basic and Translational Medicine, Xi’an Medical University, Xi’an, China; ^2^ Shaanxi Key Laboratory of Chinese Herb and Natural Drug Development, Medicine Research Institute, Shaanxi Pharmaceutical Holding Group Co., Ltd., Xi’an, China

**Keywords:** atherosclerosis, berberine, molecular mechanism, cell targets, gut microbiota

## Abstract

Cardiovascular diseases remain the leading cause of morbidity and mortality worldwide. Atherosclerosis is the main pathological basis of cardiovascular diseases and it is closely associated with hyperlipidemia, endothelial injury, macrophage-derived foam cells formation, proliferation and migration of vascular smooth muscle cells (VSMCs), platelet aggregation, and altered gut microbiota. Various symptomatic treatments, that are currently used to inhibit atherosclerosis, need to be administered in long term and their adverse effects cannot be ignored. Berberine (BBR) has beneficial effects on atherosclerosis through regulating multiple aspects of its progression. This review highlights the recent advances in understanding the anti-atherosclerosis mechanism of BBR. BBR alleviated atherosclerosis by attenuation of dyslipidemia, correction of endothelial dysfunction, inhibition of macrophage inflammation and foam cell formation, activation of macrophage autophagy, regulation of the proliferation and migration of VSMCs, attenuation of platelet aggregation, and modulation of gut microbiota. This review would provide a modern scientific perspective to further understanding the molecular mechanism of BBR attenuating atherosclerosis and supply new ideas for atherosclerosis management.

## Highlights

1) Berberine attenuated atherosclerosis by regulating dyslipidemia.
2) Berberine alleviated atherosclerosis by affecting cellular targets, including ameliorating endothelial injury, inhibiting the formation of macrophage-derived foam cells, regulating the proliferation and migration of vascular smooth muscle cells, and suppressing platelet aggregation.
3) Berberine restrained atherosclerosis by modulating gut microbiota.

## Introduction

According to the World Health Organization (WHO), an estimated 17.9 million people died of cardiovascular diseases, accounting for 30% of the total mortality worldwide (WHO, 2020). Atherosclerosis is the main pathological basis of cardiovascular diseases (Benjamin et al., 2019). The complex pathological mechanisms are developed by various factors, such as hyperlipidemia, endothelial injury, macrophage-derived foam cells formation, proliferation and migration of vascular smooth muscle cells (VSMCs), platelet aggregation, and altered gut microbiota (Tabas et al., 2015; Jonsson and Backhed, 2017; Fang et al., 2018; Qiao and Chen, 2018; Marchio et al., 2019). Atherosclerosis is initiated primarily by the accumulation of low-density lipoprotein cholesterol (LDL-C) in the vessel wall and subsequently intensified by oxidized low-density lipoprotein (oxLDL) (Marchio et al., 2019). Circulating oxLDL, increased chemokines together with the expression of adhesion proteins trigger the recruitment of immune cells, particularly monocytes (Buckley and Ramji, 2015). The monocytes then differentiate into macrophages, which engulf oxLDL and lead to foam cell formation—the hallmark of atherosclerosis (McLaren et al., 2011; Buckley and Ramji, 2015; Tabas and Bornfeldt, 2016). Subsequently, necrosis or apoptosis of foam cells, proliferation and migration of VSMCs coupled with chronic inflammatory response result in lesion development and atherosclerosis complications (McLaren et al., 2011; Buckley and Ramji, 2015; Basatemur et al., 2019).

Clinically, drugs used for symptomatic treatment mainly include lipid-lowering drugs (statins and niacins), antiplatelet and thrombolytic drugs (aspirin and urokinase), and anticoagulant drugs (warfarin). For atherosclerosis patients with ischemic symptoms, treatment of vasodilators and β-blockers such as phentolamine and propranolol can also be applied. Atherosclerosis can be effectively attenuated by these drugs, but the adverse effects of the drugs have been widely documented after long-term therapy. For example, statins can cause liver injury, myopathy, and rhabdomyolysis that cannot be ignored and there is an urgent need to develop new therapies (Björnsson, 2017; Liu et al., 2019).

**FIGURE 1:**
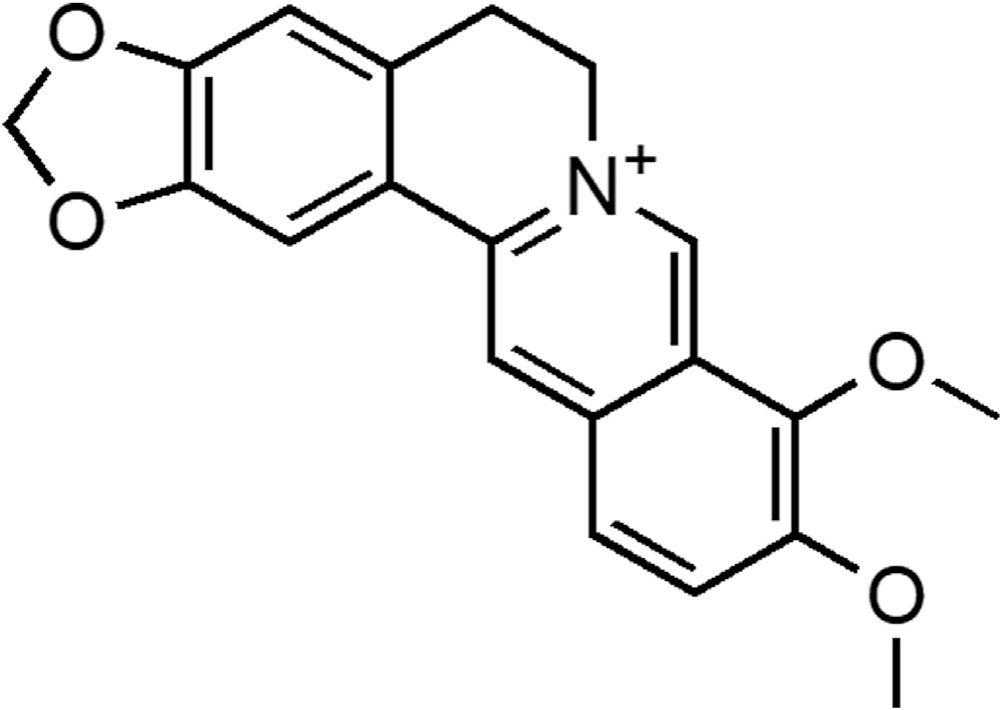
The molecular structure of Berberine.

**FIGURE 2:**
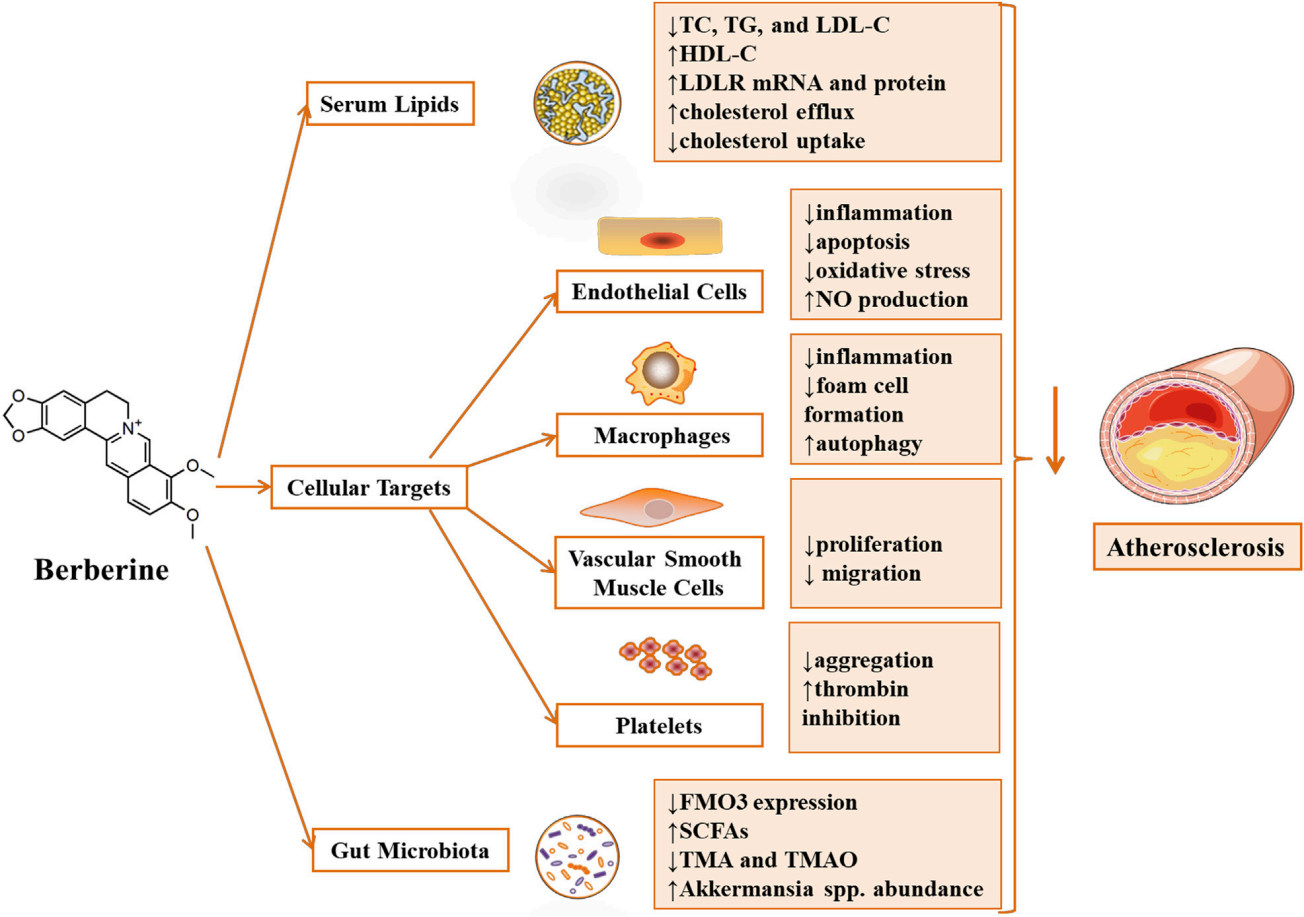
Atheroprotective effect and key molecular mechanism of Berberine (Fang et al., 2018). Berberine attenuated atherosclerosis by regulating dyslipidemia and gut microbiota. Meanwhile, Berberine alleviated atherosclerosis by affecting cellular targets, including ameliorating endothelial injury, inhibiting the formation of foam cells derived from macrophages, regulating the proliferation and migration of vascular smooth muscle cells and suppressing platelet aggregation. Annotations: ↓, reduction/down-regulation/inactivation; ↑, induction/up-regulation/activation.

The Nobel Prize in Physiology or Medicine in 2015 was awarded to Youyou Tu for the discovery of qinghaosu (artemisinin) and to William C. Campbell and Satoshi Omura for ivermectin’s discovery. This heralded a new golden age of natural product drug discovery (Li and Lou, 2018; Shen, 2015). Berberine (BBR, Figure 1) has beneficial effects on atherosclerosis through regulating multiple aspects of its progression (Neag et al., 2018; Feng et al., 2019). The guideline from the European Society of Cardiology and European Atherosclerosis Society suggested BBR as a dietary supplement and functional food for the treatment of dyslipidemia (Catapano et al., 2016). This review highlights the recent advances in understanding the anti-atherosclerosis mechanism of BBR, as shown in Figure 2. BBR alleviated atherosclerosis by attenuation of dyslipidemia, correction of endothelial dysfunction, inhibition of macrophage inflammation and foam cell formation, activation of macrophage autophagy, regulation of the proliferation and migration of VSMCs, attenuation of platelet aggregation, and modulation of gut microbiota. This review would provide a modern scientific perspective to further understanding the molecular mechanism of BBR attenuating atherosclerosis and supply new ideas for atherosclerosis management.

## Berberine Attenuated Atherosclerosis by Regulating Dyslipidemia

Hyperlipidemia, characterized by declined high-density lipoprotein (HDL) and increased total cholesterol (TC), triglyceride (TG), and LDL-C levels in serum, is a major risk factor of atherosclerosis. LDL-C plays a primary role in the formation of atherosclerosis plaque (Botham and Wheeler-Jones, 2013; Marchio et al., 2019). With the growing use of alternative herbal medicines for atherosclerosis management, BBR, as a bright new star, could alleviate atherosclerosis through regulating serum lipid profile.

According to the studies of Kong et al., orally administered BBR reduced the serum TC, TG, and LDL-C in hypercholesterolemic patients after a 3-months treatment. BBR activated extracellular signal-regulated kinase (ERK) and increased the mRNA stability of low-density lipoprotein receptor (LDLR), thus exhibited lipid-lowering effects in hyperlipidemic hamsters and HepG2 cells (Kong et al., 2004). This finding is consistent with a recent study conducted by Zhou et al., who suggested that BBR and its metabolites increased the LDLR mRNA and protein and had beneficial effects on inhibiting cellular lipid accumulation (Zhou et al., 2014). Clinical trials indicated that BBR increased plasma HDL-C and reduced TC, TG, and LDL-C after three months of administration (1.0 g daily) in subjects with low cardiovascular risk and patients with dyslipidemia and type 2 diabetes (Zhang et al., 2008; Derosa et al., 2013). The combination of BBR and simvastatin reduced serum LDL-C (46.2%) more effectively than that of BBR (26.8%) or simvastatin (28.3%) administered alone. This role might be attributed to the up-regulatory effects on LDLR expression of BBR, which is distinct from the inhibition of 3-hydroxy-3-methylglutaryl-coenzyme A reductase with statins (Kong et al., 2008). Another study by Brusq et al. demonstrated that BBR inhibited lipid synthesis in HepG2 cells through the activation of adenosine monophosphate-activated protein kinase (AMPK) in addition to upregulating the LDLR (Brusq et al., 2006). Recent studies showed that BBR could alleviate hyperlipidemia partly by promoting intracellular cholesterol efflux and decreasing cholesterol uptake by enterocytes (Wang et al., 2014; Li et al., 2015; Ma et al., 2020a).

## Berberine Alleviated Atherosclerosis by Affecting Cellular Targets

### Endothelial Cells

Vascular endothelium, the inner layer of the cardiovascular system, is a major regulator of vascular homeostasis in healthy individuals (Gimbrone and Garcia-Cardena, 2016). The healthy endothelium function mainly as a mechanical barrier between blood vessel walls and plasma molecules. Besides, it can respond to physical and chemical stimuli by producing numerous factors that regulate leukocyte attachment, vascular tone, thromboresistance, vessel wall inflammation, and VSMCs proliferation (Deanfield et al., 2007). Endothelial cell dysfunction plays a vital role in atherosclerosis lesion initiation and progression.

#### Berberine Suppressed Endothelial Proinflammation

A spectrum of factors lead to endothelial dysfunction, which results in the expression of endothelial-leukocyte adhesion molecules [e.g., vascular cell adhesion molecule-1 (VCAM-1), intercellular adhesion molecule-1 (ICAM-1), and endothelial-leukocyte adhesion molecule-1], secreted chemokines [e.g., monocyte chemoattractant protein-1 (MCP-1), interleukin-8 (IL-8)] and other effector proteins (Gimbrone and Garcia-Cardena, 2016). These events bring about the recruitment of numerous inflammatory cells and trigger vascular inflammation.

BBR was reported to dramatically decrease oxLDL-stimulated adhesion of monocytes to human umbilical vein endothelial cells (HUVECs) by suppressing the expression of VCAM-1 and ICAM-1 (Huang et al., 2013). The results from Wang et al. showed that BBR attenuated the production of adhesion molecules and suppressed monocyte attachment to endothelial cells. Therefore, the hyperglycemia-induced endothelial injury was prevented partly by activating the AMPK signaling cascade (Wang et al., 2009b). Ko et al. revealed that BBR dose-dependently suppressed angiotensin II-induced U937 cells adhesion to HUVECs and mRNA expression of C-C chemokine receptor 2 (CCR-2) in U937 monocytes and MCP-1 in HUVECs, thus effectively alleviated angiotensin II-induced endothelial inflammation (Ko et al., 2007). HMC05, an extract containing BBR, inhibited attachment of monocytes to endothelial cells dose-dependently via decreasing the levels of VCAM-1, ICAM-1, MCP-1, and CCR-2 after tumor necrosis factor-α (TNF-α) induction, which was similar to that of BBR (Lee et al., 2011).

#### Berberine Inhibited Endothelial Cell Apoptosis

Apoptosis of vascular endothelial cells contributes to atherosclerosis development. The endothelial cells undergo apoptosis when exposed to various environmental changes, such as elevated oxLDL, blood glucose, and reactive oxygen species (ROS), decreased nitric oxide, and low shear stress (Paone et al., 2019).

BBR down-regulated the expression of proliferating cell nuclear antigen, nuclear factor κB (NF-κB), and lectin-like oxLDL receptor-1. Meanwhile, BBR inactivated phosphatidylinositol 3 kinase (PI3K)/AKT serine/threonine kinase (Akt), ERK1/2, and p38 mitogen-activated-protein kinase (MAPK) signaling pathways. Thus, BBR protected against oxLDL-caused endothelial dysfunction (Wang et al., 2009b; Caliceti et al., 2017; Xu et al., 2017). Pretreatment of BBR suppressed lipopolysaccharide (LPS)-induced apoptosis in HUVECs by blocking the c-Jun N-terminal kinase-mediated signaling pathway (Guo et al., 2016). BBR also alleviated high-glucose-mediated endothelial damage and enhanced vasodilatation *via* activating AMPK signaling cascade (Wang et al., 2009b).

#### Berberine Attenuated Oxidative Stress

Oxidative stress is the imbalance of excessive ROS generation and inactivated antioxidant defense systems. ROS generators in the vessel wall include nicotinamide adenine dinucleotide phosphate (NADPH) oxidase, xanthine oxidase, mitochondrial enzymes, and uncoupled endothelial nitric oxide synthase (eNOS). The antioxidant enzymes in atherosclerosis contain superoxide dismutase, catalase, glutathione peroxidase, and paraoxonases (Förstermann et al., 2017).

BBR treatment ameliorated CD31^+^/CD42^−^ microparticles-induced endothelial dysfunction through decreasing oxidative stress in HUVECs (Cheng et al., 2013). Studies conducted by Wang et al. (2009b), Zhang et al. (2013) demonstrated that BBR alleviated endothelial injury induced by high glucose and palmitate partly via activation of the AMPK signaling cascade and reduced generation of ROS. BBR could reduce intracellular ROS levels induced by TNF-α (Caliceti et al., 2017) and endothelial progenitor cells dysfunction caused by TNF-α could be improved by BBR *via* PI3K/Akt/eNOS signal pathway (Xiao et al., 2014). Furthermore, HMC05, an extract containing BBR, markedly inhibited the production of ROS and dose-dependently attenuated TNF-α-induced adhesion of monocytes to endothelial cells (Lee et al., 2011).

#### Berberine Activated Nitric Oxide Signaling Pathway

Nitric oxide (NO) produced by nitric oxide synthase (NOS) in endothelial cells is of great importance in regulating vascular tone. Neuronal NOS, eNOS, and inducible NOS are related to the production of NO. Neuronal NOS and eNOS function as anti-atherosclerosis factors, whereas inducible NOS is likely to play a pro-atherosclerosis role (Li et al., 2014). BBR showed atheroprotective effects by affecting the NO signaling pathway.

It was demonstrated that phosphorylation of eNOS at Ser1177 was enhanced by BBR dose-dependently, leading to an increased eNOS protein expression and NO production (Wang et al., 2009b). Zhang et al. (2013) reported that BBR considerably upregulated eNOS expression and NO levels in palmitate-treated HUVECs and ameliorated endothelial dysfunction. Bu-Shen-Ning-Xin Decoction, a Chinese herbal compound containing BBR, upregulated NO synthesis via estrogen receptor *β* pathway. Subsequently, NO suppressed apoptosis and NF-κB activity in endothelial cells and inhibited atherosclerosis progression (Wang et al., 2013). Elevated circulating endothelial microparticles (EMPs) are tightly linked to endothelial dysfunction. The diminished eNOS protein expression mediated by EMPs was markedly inhibited by BBR in HUVECs. Furthermore, BBR-induced decline in circulating CD31^+^/CD42^−^ microparticles contributed to the improvement of endothelial function in healthy subjects (Wang et al., 2009a; Cheng et al., 2013).

### Macrophages

Macrophages play critical roles in the initiation and progression of atherosclerosis. The inflammatory responses and macrophage-derived foam cell formation are the principal events in atherosclerosis (Moore et al., 2013; Tabas and Bornfeldt, 2016). BBR can achieve its atheroprotective functions by affecting the behavior of macrophages, such as inhibition of macrophage inflammation, foam cell formation, and activation of macrophage autophagy.

#### Anti-Inflammation

Macrophages constitute the most prominent inflammatory cells in atherosclerosis lesions. Activated macrophages produce a series of inflammation-related factors such as interleukin-1β (IL-1β), TNF-α, interleukin-6 (IL-6), IL-8, MCP-1, matrix metalloprotease-9 (MMP-9), and so on, which initiate inflammation to induce atherosclerosis (Kleemann et al., 2008).

BBR significantly downregulated the expression of proinflammatory genes such as IL-1β, IL-6, MCP-1, inducible NOS, cyclooxygenase-2, and MMP-9 through AMPK activation in macrophages (Jeong et al., 2009). In oxLDL-induced macrophages, BBR markedly upregulated miR150-5p level and decreased P2X7R-mediated extracellular matrix metalloproteinase inducer (EMMPRIN) and MMP-9 expression (Lu et al., 2021). In LPS-stimulated macrophages (RAW264.7), BBR treatment potently suppressed the expression of inflammatory cytokines such as TNF-α, IL-6, and MCP-1 through inhibition of NF-κB signaling *via* sirtuin 1-dependent mechanisms (Zhang et al., 2017). According to the study by Chen et al., BBR inhibited acetylated low-density lipoprotein-induced TNF-α, MCP-1, and IL-6 expression through peroxisome proliferator-activated receptor *γ* signaling pathway in macrophages (Chen et al., 2008). BBR tremendously inhibited TNF-α and IL-6 expression stimulated with an HIV protease inhibitor by modulating endoplasmic reticulum stress signaling pathways in murine macrophages (Zha et al., 2010). BBR reduced the expression of MMP-9 and EMMPRIN by suppressing the activation of p38 and NF-κB signaling pathways in human THP-1 macrophages (Huang et al., 2011; Huang et al., 2012b). BBR alleviated NLR Family Pyrin Domain Containing 3 inflammation activation by reducing IL-1β secretion *via* NF-κB inhibition in macrophages (Jiang et al., 2017). HMCO5 containing BBR suppressed the activation of NF-κB and subsequently inhibited the secretion of TNF-α and IL-1β in LPS stimulated RAW264.7 cells (Kim et al., 2007). In mouse RAW264.7 macrophages and primary hepatocytes, BBR significantly downregulated the proinflammatory cytokines (TNF-α, IL-6, IL-1β, and MCP-1) *via* suppressing the protein expression of endoplasmic reticulum stress genes (Wang et al., 2020b).

#### Berberine Inhibited Foam Cell Formation

Foam cell formation is a hallmark at the initial stage of atherosclerosis. The augmented ox-LDL influx and accumulation of cholesterol esters in intimal macrophages are responsible for this issue. Macrophages express a series of scavenger receptors (SR) with affinity to oxLDL, such as SR class A type I, CD36, and LOX-1. ATP-binding cassette transporters ABCA1 and ABCG1 and SR class B type I (SR-BI) in macrophages are involved in reverse cholesterol transport (Chistiakov et al., 2016; Chistiakov et al., 2017). These proteins protected macrophages from the formation of foam cells.

BBR can dose- and time-dependently downregulate oxLDL receptor-1 expression and facilitate SR-BI expression in macrophage-derived foam cells induced by oxLDL (Guan et al., 2010). Simultaneous administration of BBR and atorvastatin inhibited the expression of LOX-1 *via* the endothelin-1 receptor in monocyte/macrophages, which inhibited foam cell formation (Chi et al., 2014). BBR reduced foam cell formation by decreasing oxLDL internalization and increasing cholesterol efflux *via* the suppression of CD36, lectin-like oxLDL receptor-1, and adipocyte enhancer binding protein 1 in macrophages (Huang et al., 2012a). Macropinocytosis, excess free cholesterol-induced membrane ruffling, and hypercholesterolemic serum-induced cholesterol accumulation were inhibited by BBR in macrophages (Zimetti et al., 2015). BBR inhibited foam cell formation by increasing cholesterol efflux through enhancing liver X receptor α-ABCA1 expression in macrophages (Lee et al., 2010).

#### Berberine Promoted Macrophage Autophagy

Macrophage autophagy inhibited foam cell formation by the deficiency of oxLDL ingestion and the increase of efferocytosis and cholesterol efflux in macrophages. Therefore, promoting macrophage autophagy may alleviate atherosclerosis (Jia et al., 2006; Muller et al., 2011; Scherz-Shouval and Elazar, 2011; Shao et al., 2016).

BBR treatment alleviated inflammation in murine macrophages (J774A.1) by promoting autophagy, which was initiated by activation of the AMPK/mechanistic target of rapamycin (mTOR) signaling pathway (Fan et al., 2015). BBR-mediated sonodynamic therapy effectively induced cholesterol efflux by promoting ROS generation, and induced autophagy by regulating the PI3K/Akt/mTOR signaling pathway in THP-1 macrophages, peritoneal macrophages, and derived foam cells (Kou et al., 2017). BBR activated Sirt1 *via* the nicotinamide adenine dinucleotide synthesis pathway to promote transcription factor EB nuclear translocation and deacetylation, which in turn, triggered autophagy in peritoneal macrophages (Zheng et al., 2021). BBR reduced plaque area and alleviated inflammation in atherosclerosis rats with damp-heat syndrome *via* promoting LC3-II protein expression and inhibiting P62 protein expression. 3-methyladenine, an inhibitor of autophagy, significantly aggravated atherosclerosis progression (Ke et al., 2020).

### Vascular Smooth Muscle Cells

VSMCs play a critical role in atherosclerosis progression. The aberrant proliferation and migration of VSMCs promote extracellular matrix formation in atherosclerosis plaque areas (Doran et al., 2008; Chistiakov et al., 2015). Studies confirmed that BBR could suppress the proliferation and migration of VSMCs to attenuate atherosclerosis.

Angiotensin II and heparin-binding epidermal growth factor were enormously inhibited by BBR *via* delaying or partially inactivating the Akt signaling pathway, which inhibited the proliferation and migration of VSMCs (Lee et al., 2006). Lysophosphatidylcholine induced VSMCs proliferation and migration, which triggered the intimal thickening in atherosclerosis lesions. BBR inhibited lysophosphatidylcholine-stimulated VSMCs proliferation and migration *via* suppression of ROS generation and ERK1/2 signaling pathway (Cho et al., 2005). BBR inhibited platelet-derived growth factor (PDGF)-induced VSMCs growth *via* activation of AMPK/p53/p21^Cip1^ signaling pathway and suppressed PDGF-stimulated migration *via* inhibition of Ras, Cell Division Cycle 42, and Rac Family Small GTPase 1 (Liang et al., 2008). Mechanical injury-induced VSMCs growth was prevented by BBR treatment through mitogen-activated protein kinase/ERK activation, early growth response gene, c-Fos, Cyclin D1, and PDGF subunit A expression, protein disulfide isomerase activation as well as phosphorylation of MAPKs (Liang et al., 2006; Wang et al., 2020a). BBR disrupted the binding of p27, p21 with S-phase kinase-associated protein-2, and induced G0/G1 phase arrest, which attenuated the proliferation of A7r5 induced by PDGF (Liu et al., 2011). Liu et al. found that BBR exerted anti-migratory properties in human VSMCs, possibly by downregulating MMP-2/9 and urokinase-type plasminogen activator and inhibiting AP-1 and NF-κB signaling pathways (Liu et al., 2014). BBR treatment dose-dependently inhibited VSMCs migration induced by upregulations of MMP-3 and MMP-9 *via* decreasing the phosphorylation of Akt at Ser473 with *C. pneumoniae* infection (Ma et al., 2015). HMC05, containing BBR and hesperidin in large quantities, protected VSMCs against oxidative stress by increasing NADPH: quinone oxidoreductase-1 gene expression *via* the regulation of Ras homolog family member A and/or Ras (Gum et al., 2014).

### Platelets

Impaired regulation of platelet activation/aggregation is a prime cause of arterial thrombosis, this vital complication of atherosclerosis triggering myocardial infarction and stroke (Schafer and Bauersachs, 2008). The platelet activation and apoptosis would induce vascular occlusions and atherothrombotic events. BBR could inhibit these events by suppressing platelet aggregation and superoxide production *via* regulating NADPH oxidase, aldose reductase, and glutathione reductase in platelets with excess glucose. In addition, BBR inhibited platelet adhesive property and apoptosis induced by high glucose (Paul et al., 2019). BBR significantly inhibited rabbit platelet aggregation by suppressing the synthesis of thromboxane A2 (Huang et al., 2002). Molecular docking studies indicated that BBR interacted with thrombin by hydrogen bond and π-π interactions. Direct binding studies, competitive binding assay, and platelet aggregation assay demonstrated that BBR was a thrombin inhibitor showing direct activity in inhibiting platelet aggregation (Wang et al., 2017).

## Berberine Reduced Atherosclerosis by Affecting Gut Microbiota

The gut microbiota and its metabolites play a critical role in atherosclerosis development (Mantziaris and Kolios, 2019). Trimethylamine (TMA), produced by gut microbiota, was converted to trimethylamine-N-oxide (TMAO) *via* flavin-containing monooxygenase form 3 (FMO3) in the liver (Schiattarella et al., 2017; Mantziaris and Kolios, 2019; Tang et al., 2019). It has been found that the BBR treatment reduced high-fat diet feeding-induced FMO3 expression and altered the composition of gut microbiota (Shi et al., 2018). The synthesis of TMA and TMAO were inhibited remarkably in choline-fed ApoE^-/-^ and C57BL/6J mice by BBR *via* suppressing choline-to-TMA conversion. However, a slight increment was observed in chow-fed mice, indicating that BBR might decrease TMA production by gut microbiota only when the choline was overdosed (Li et al., 2021). There was a piece of evidence that BBR directly changed the bacterial community composition and function by reducing *Clostridium* spp. and subsequently activated farnesoid X receptor signaling (Tian et al., 2019). BBR treatment markedly increased *Akkermansia* spp. abundance in HFD-fed ApoE^-/-^ mice, contributing to the anti-atherosclerotic properties of BBR (Zhu et al., 2018). In line with those findings, replenishment with *Akkermansia* significantly reduced atherosclerosis induced by a high-fat diet by attenuating the aortic and systemic metabolic inflammatory response (Li et al., 2016). A previous study revealed that BBR stimulated the gut bacteria-derived polyamines and enhanced mucin secretion in the colon of mice, exhibiting *Akkermansia*-promoting effects (Dong et al., 2021). According to the study of Wu et al., the abundance of *Alistipes*, *Allobaculum*, *Blautia*, *Roseburia*, and *Turicibacter* were significantly increased, and the abundance of *Bilophila* was altered after BBR treatment. Thus, the metabolism of lipid, glycan and the synthesis of short-chain fatty acids were promoted and the production of TMAO was reduced (Wu et al., 2020).

## Concluding Remarks

Herbal medicines represent indispensable roles in new drug discovery, and they are relatively safe since herbs have been used for thousands of years in clinical practice. The atheroprotective effects of BBR have been explored during the past decades. We reviewed its anti-atherosclerotic effects from the perspective of molecular targets. Numerous evidences suggested that BBR had great therapeutic potential to attenuate atherosclerosis through lipid modification, anti-inflammatory, anti-oxidant, anti-apoptosis, anti-proliferative, anti-platelet aggregation, and gut microbiota modulatory activities. Among them, anti-inflammatory was the dominant factor. BBR significantly inhibited the expression of inflammatory factors and adhesion molecules, thus played anti-inflammatory role both in macrophages and endothelial cells.

Although a lot of knowledge has been gained in understanding the BBR-mediated atheroprotective potential, there are numerous questions ahead. The poor aqueous solubility and low dissolution of BBR lead to low oral bioavailability (< 1%) and have limited its clinical application (Liu et al., 2010). However, the poor bioavailability of BBR and its favorable atheroprotective effects are not contradictory. On the one hand, poorly absorbed BBR remained inside the gastrointestinal tract for a long time. It interacted comprehensively with the gut microbiota, which contributed to the anti-atherosclerosis effects of BBR by regulating the gut microbiota. On the other hand, BBR could convert into multiple metabolites. Many metabolites have anti-atherosclerotic effects, some metabolites showed even more potent anti-atherosclerotic effects than BBR (Cho, 2011; Cao et al., 2013; Wu et al., 2014; Zhou et al., 2014; Ning et al., 2015). In addition, various approaches have been explored to enhance its oral bioavailability (Mujtaba et al., 2021). BBR-trapped solid lipid nanoparticles and micelles had shown anti-hyperlipidemic and anti-atherosclerosis effects in animals (Ma et al., 2020b; Sailor et al., 2021). Some BBR analogs and derivatives also exhibited anti-atherosclerosis properties (Feng et al., 2017a; Feng et al., 2017b). Our understanding of BBR has been deepening by chemical, pharmacological, and system biological approaches (Liu et al., 2013). Especially, with the help of network pharmacology, computer-assisted molecular docking and genomic, and metabolomic profiling approaches, novel anti-atherosclerosis mechanisms/targets of BBR will be identified. In short, BBR could be a promising candidate for atherosclerosis management.

## Glossary

Akt: AKT serine/threonine kinase
AMPK: adenosine monophosphate-activated protein kinase
BBR: berberine
CCR-2: C-C chemokine receptor 2
EMPs: endothelial microparticles
eNOS: endothelial nitric oxide synthase
ERK: extracellular signal-regulated kinase
HDL: high-density lipoprotein
HUVECs: human umbilical vein endothelial cells
ICAM-1: intercellular adhesion molecule-1
IL-8: interleukin-8
IL-1β: interleukin-1β
IL-6: interleukin-6
LDL-C: low-density lipoprotein cholesterol
LDLR: low-density lipoprotein receptor
LPS: lipopolysaccharide
MAPK: mitogen-activated-protein kinase
MCP-1: monocyte chemoattractant protein-1
MMP-9: matrix metalloprotease-9
NADPH: nicotinamide adenine dinucleotide phosphate
NF-κB: nuclear factor κB
NOS: nitric oxide synthase
oxLDL: oxidized low-density lipoprotein
PCNA: proliferating cell nuclear antigen
PDGF: platelet-derived growth factor
PI3K: phosphatidylinositol 3 kinase
ROS: reactive oxygen species
SR: scavenger receptors
TC: total cholesterol
TG: triglyceride
TMA: trimethylamine
TMAO: trimethylamine-N-oxide
TNF-α: tumor necrosis factor-alpha
VCAM-1: vascular cell adhesion molecule-1
VMSCs: vascular smooth muscle cells
WHO: World Health Organization
